# How to measure ego-resiliency in the face of various life-changing crises: Measurement invariance, convergent and discriminant validity and reliability of the Polish version of the Revised Ego-Resiliency Scale (ER89-R12)

**DOI:** 10.7717/peerj.14499

**Published:** 2023-01-09

**Authors:** Anna Kołodziej-Zaleska, Michalina Ilska, Anna Brandt-Salmeri, Anna Jazłowska, Hanna Przybyła-Basista

**Affiliations:** 1 Institute of Psychology, University of Silesia, Katowice, Poland; 2 University of Economics and Human Sciences in Warsaw, Warsaw, Poland

**Keywords:** Resilience, Ego-resiliency, Crisis, Mental health, Measurement invariance, Psychological well-being, Cancer, Down’s syndrome, Divorce, Stress

## Abstract

This study examines the generalizability of the latent structure of the Polish version of the Ego-Resiliency Scale (ER89-R12), a brief self-report scale that measures ego-resiliency. We investigated the measurement invariance, validity, and reliability of ER89-R12 among three groups of individuals who were facing various major, long-term, life-changing crises (*N* = 512): parents of children with Down’s syndrome, women with breast cancer, and individuals after divorce. The analysis of the measurement invariance confirmed the two-factor structure of the questionnaire and the high reliability of this measure in those studied groups. A multigroup confirmatory factor analysis provided evidence of configural, metric, scalar, and residual invariance across the three groups. Moreover, the correlation patterns were similar across the groups. Ego-resiliency was strongly and consistently positively correlated with mental health: psychological well-being, perceived social support, self-esteem, and post-traumatic growth, and negatively correlated with perceived stress. The presented results indicate the potential usefulness of the ER89-R12 tool in studies on people experiencing various crises in their lives.

## Introduction

In recent years, there has been a noticeable increase in interest in resilience and a surge in the number of studies devoted to this issue. Many of these studies have aimed to establish the definitional or conceptual meaning of resilience and to review the available research tools (Arat, 2014; Fletcher & Sarkar, 2013; Windle, Bennett & Noyes, 2011). This area of research is characterized by high complexity in terms of definitions, with variously-defined concepts used throughout the literature: ‘resilience’, ‘resiliency’, and the most semantically clarified concept, ‘ego-resiliency’. Definitions of the terms ‘resilience’ and ‘resiliency’ are most often built around two key constructs: adversity and positive adaptation (Fletcher & Sarkar, 2013). While the term ‘resilience’ refers to the dynamic process responsible for effective adaptation in unfavourable life circumstances (Bonanno, 2004; Luthar, Cicchetti & Becker, 2000), ‘resiliency’ refers to a relatively constant personality trait that facilitates coping with stress effectively (Bonanno & Mancini, 2012). This characteristic allows for flexible adaptation and the mobilization of resources, which increases tolerance to negative emotions (Ong et al., 2006). People with a high level of resilience are characterized by emotional stability, a tendency towards the positive over the negative, and a greater sense of their own efficacy (Tugade & Fredrickson, 2004). They tend to interpret unfavourable circumstances in terms of challenges and opportunities for new experiences, rather than burdens (Tugade & Fredrickson, 2004; Ogińska-Bulik, 2013). Above all, the features of resilience are revealed in the way a person adjusts following exposure to a highly stressful event. This is manifested through stable behavioural patterns and the ability to maintain balance (Bonanno, 2004). The key concepts of resilience are flexible adaptation and effective coping. A person who is resilient shows a significantly higher level of capacity for positive and resourceful adaptation to external and internal stressors (Klohnen, 1996; Alessandri et al., 2012). Therefore, he does not experience symptoms of post-traumatic stress due to the protective nature of his or her resilience resilience (Bonanno, 2004; Bonanno & Mancini, 2012). Moreover, resilient people return to their pre-crisis state more quickly, and have a better mental health (Lepore & Revenson, 2006).

The definitive and conceptual complexity of the analysed concepts, and thus the tools used to measure the studied variables, results mainly from the fact that there are three different issues involved in resilience (Luthar, 2015; Windle, Bennett & Noyes, 2011). Firstly, resilience is a process requiring specific resources or protective factors, which leads to a specific result. Secondly, resilience can be seen as the actual result of this process. In this case, researchers are interested in the resources—personal and family, environmental and, more broadly, cultural—that facilitate resilience. Thirdly, resilience is also the individual ability to return to balance (bounce back), cope with adversities, adapt flexibly, and so on (Windle, Bennett & Noyes, 2011). In the latter sense, the terms ‘resiliency’ or ‘ego-resiliency’ are often used, although some researchers use the term ‘resilience’ to mean the disposition, not the process or its result (Bonanno, 2004; Kowalczyk et al., 2015; Perrig-Chiello, Hutchison & Morselli, 2015; Quinney & Fouts, 2004). Interestingly, for Bonanno (2004), resilience is more an individual trait than a process.

### Ego-resiliency as a personality trait

The concept of ego-resiliency was proposed by Block & Block (1980) to define a constellation of features that favour coping with adversities, equating the concept of ego-resiliency with the trait of resilience—that is, an individual capacity for rapid and adequate self-regulation. It can be said that links exist between resilience and ego-resiliency. The ego-resiliency trait allows resilient individuals—those people with high ego-resiliency—to adapt faster and more satisfactorily to changing circumstances (Block & Kremen, 1996). Ego-resiliency can be treated as a protective factor in the resilience process. However, it would be inappropriate to use this measure as a determinant of resilience (Windle, Bennett & Noyes, 2011). Ego-resiliency as the only determinant of the resilience process is insufficient.

According to Block & Kremen (1996), ego-resiliency is one of the basic personality traits that enables the understanding of motivational, emotional and behavioural processes, the construct that forms the foundation of personality theories. Ego-resiliency is a person’s dynamic ability to constantly modify the level of control resulting from interactions between the individual and their environment (Block & Block, 1980; Block & Kremen, 1996). Excessive ego-control is associated with a high threshold of impulse expression and a tendency to internalize problems, depression and anxiety (see: Vanderbleek & Kirsten, 2018). On the other hand, a deficiency in ego-control is associated with a low threshold of impulse expression and may manifest itself in externalization problems: crime, aggression, hyperactivity, or excessive impulsiveness (Block & Block, 1980; Letzring, Block & Funder, 2005).

In the broadest sense, ego-resiliency is the overall ability to adapt to external and internal stressors in a flexible and satisfactory way. It enables the dynamic maintenance of balance between emotional rigidity and excessive impulsiveness. Letzring and colleagues (2005) equate ego-resiliency with the ability to contextually modify ego-control in response to adversity. They describe it as a ‘meta-dimension’ that allows for an adequate assessment of the adaptation requirements posed by a difficult situation. It refers not only to a specific way of functioning, but also to a specific personality structure characterized by a higher level of organization (Farkas & Orosz, 2015). It can therefore be said that ego-resiliency is a kind of meta-trait related to ‘higher-level organization’, as it refers to the flexibility and adaptability of the personality as a whole (Farkas & Orosz, 2015).

### The structure of ego-resiliency

According to Klohnen (1996) too little attention is paid to explaining both the conceptual meaning and the components of resilience, understood as a variable in the field of individual differences. In terms of ego-resiliency as a differentiating trait, knowledge of its components would help us to understand why and how some people satisfactorily adapt to changing or even threatening circumstances, while others surrender, suffer, or experience illness, disorders, mental problems and crises. Ego-resiliency is considered as an overarching personality resource consisting of interrelated features like: trusting optimism, productive, creative and autonomous activity, interpersonal warmth, insight/intuition, and the ability to be expressive in social interactions (Klohnen, 1996).

The authors of the scale for measuring the ego-resiliency construct (Block & Kremen, 1996) assumed it to be unidimensional; thus, their tool had a single-factor structure. From the perspective of our research, important findings have been made by Italian researchers (Alessandri et al., 2012; Vecchione et al., 2010). Their studies suggest more complex than a single-factor structure for the scale used to measure ego-resiliency. Alessandri et al. (2007) claim that there is an issue with the dimensionality of the ER89 Ego-Resiliency Scale. The same problem was discussed in earlier studies of Menesini & Fonzi (2005, after Alessandri et al., 2007), in which the researchers question the unidimensionality of the ER89 Scale and suggest two factors: Resiliency-Self Regulation, which refers to the items related to agreeableness and the ability to self-regulate, and Resiliency-Openness, which refers to a group of statements denoting openness and curiosity. Research conducted by Alessandri et al. (2007) confirmed that the empirical evidence for the unidimensionality of the ego-resiliency construct is insufficient. Based on indices of fit, they showed that a two-factor structure is more reliable.

The two ego-resiliency components they distinguished were already reflected in Block and Block’s theoretical considerations, specifically in their theory of ego-resiliency (Block & Block, 1980). This is one of the reasons why Alessandri et al. (2007) named the two factors ‘Optimal Regulation’ and ‘Openness to Life Experience’. According to Alessandri et al. (2007), these two factors comprise the overall dimension of ego-resiliency. Importantly, they also showed that the components of ego-resiliency are associated with stability in the case of optimal regulation, and plasticity in the case of openness to life experience (Alessandri et al., 2007). The stability of the two-factor version of the tool was confirmed using longitudinal studies conducted on adolescents and young adults (Vecchione et al., 2010), while its reliability and validity in cross-cultural studies were shown by Alessandri et al. (2012). The Polish adaptation of the ER89 (Kołodziej-Zaleska & Przybyła-Basista, 2018a) confirms the structure of the tool proposed by the Italian researchers.

Moreover, the unidimensional nature of the ego-resiliency construct was not confirmed in Hungarian studies (Farkas & Orosz, 2015), which describe ego-resiliency as active involvement in the world, possessing a repertoire of problem-solving strategies, and being able to integrate measures under stressful conditions. Farkas & Orosz (2015) consistently demonstrate both multidimensionality in the measurement of adaptive elasticity and the fact that different aspects of ego-resiliency may function independently of each other in different circumstances.

### The ER89-R12 Scale for measuring ego-resiliency in studies of people facing crises

The Polish adaptation of the Ego-Resiliency Scale (ER89-R12) has been applied in research in different contexts and with various groups of respondents. It has been used in studies carried out in the field of the psychology of close relationships, marriage and family (Kołodziej-Zaleska, 2019; Kołodziej-Zaleska & Przybyła-Basista, 2020; Kózka & Przybyła-Basista, 2017; Kózka & Przybyła-Basista, 2018), health psychology and psycho-oncology (Brandt-Salmeri, 2020), prenatal psychology (Ilska, 2020; Ilska & Przybyła-Basista, 2020; Ilska, Brandt-Salmeri & Kołodziej-Zaleska, 2020), and personality psychology (Pyszkowska, 2020). Most of these studies have focused on searching for the personal and relational resources that contribute to developing the mental well-being of people experiencing various normative and non-normative crises. These studies used the ER89-R12 scale and assumed that ego-resiliency would be a key personal resource in the process of adaptation to a crisis situation, as well as strengthening psychological well-being and improving quality of life. There is a significant positive correlation between ego-resiliency and other resources, including self-esteem, positive orientation, psychological flexibility, and level of mental well-being, happiness, and life satisfaction; on the other hand, the correlation between ego-resiliency and the level of perceived stress, anxiety and depression, according to the assumptions, is negative (Ilska, 2020; Ilska, Brandt-Salmeri & Kołodziej-Zaleska, 2020; Kołodziej-Zaleska, 2019; Pyszkowska, 2020). In all these studies, the two-factor structure of the tool was confirmed, and the questionnaire was characterized by satisfactory statistical parameters: high reliability measured by the Cronbach’s alpha coefficient for the entire scale, and high or acceptable internal consistency for the separate subscales.

### The present study

Based on the framework delineated above, the primary objective of this study was to confirm the psychometric equivalence of a construct (ego-resiliency) across three groups of people facing different life-changing crises. The present study also examined the validity of the tool for measuring ego-resiliency in the analysed groups as well as the differences between the groups. First, it was assumed the equivalence of measurement in all study samples. Ego-resiliency would not differentiate the samples and would have the same structure. Second, it was assumed the convergent and discriminant validity, and realiability of Polish version of Revised Ego-Resiliency Scale (ER89-R12).

## Materials and Methods

### Procedure and participants

A cross-sectional study design was used. The study was conducted with three samples of individuals who were facing major, long-term crises of various natures (*N* = 512): parents of children with Down’s syndrome (sample A), women with breast cancer (sample B), and individuals after divorce (sample C). Participants were recruited in Poland from institutions that help people (foundations supporting parents of children with Down’s syndrome, diagnostic and consultation centres for families, child and family support centres, Amazon Breast Cancer Support Groups, foundations supporting breast cancer patients, and oncology departments). The research was carried out within several different projects where ego-resiliency was treated as a personality trait that might be considered a protective factor in the process of adaptation to different psychological crises.

Participants in all three samples were informed about the purpose of the study and agreed to participate. The instructions assured them of the voluntary nature of participation, as well as anonymity. All participants were informed that they could withdraw from the study at any time. Participants did not provide any personal data and a coding system was used for the questionnaires. Additionally, they returned the questionnaires in sealed envelopes.

We received informed consent from all participants in the three samples. After reading the information sheets they answered yes or no for question: Do you agree to particiate in this study? We included only the questionnaires of the participants, who answered yes. The Ethics Committees approved the study procedures in each of the three samples.

#### Parents of children with Down’s syndrome (sample A)

The participants in this group were 126 parents of children with Down’s syndrome, of whom 59.52% (*n* = 75) were mothers and 40.48% (*n* = 51) were fathers. The parents’ ages ranged from 25 to 69 years with a mean age of 46.54 years (*SD* = 10.26). All the parents were married at the time of participation in the study. The age of the children (with Down’s syndrome), of whom 52% were girls and 48% were boys, ranged from 1 to 39 years (*M* = 13.25, *SD* = 8.90). The group also included a wide range in terms of number of years married (min. = 5, max. = 48, *M* = 20.5, *SD* = 10.62). The most numerous group (almost 43%) had higher education, slightly over 36% had secondary education, and about 20% had vocational education. Half of the respondents were employed full-time. Over 80% of the surveyed parents described their financial situation as sufficient or good.

#### Women with breast cancer (sample B)

This sample consisted of 229 women with breast cancer. The average age of the women was 53.48 years (*SD* = 10.45). The vast majority of women lived in large cities (48%) and smaller towns (nearly 38%). The most numerous group were women with secondary education (47.6%), followed by women with higher education (34.1%) and women with vocational education (14.4%), and the least numerous were women with primary education (3.5%). In terms of marital status, the majority of women were married (73.4%), and the next largest group were widowed (12.2%). A total of 6% of women were in an informal relationship, 5.7% were divorced and 2.6% were single. The majority of the sample (*n* = 216) had children. Over half of the women were no longer working (62.7%); of these women, nearly 50% were retired, and nearly 16% were receiving disability pension. A total of 32.4% of the women worked part-time and only 1.7% were unemployed.

The average time from receiving their breast cancer diagnosis was 5.28 years (*SD* = 4.27). Most of the women had already completed treatment (62.4%), and 35.8% were still undergoing treatment. Regarding the stage of cancer (which corresponds to the level of malignancy of the disease), the most numerous group of women had stage II cancer (41.5%), and slightly fewer had stage III (30.6%). The most common treatment was the combined method (chemotherapy and/or hormone therapy and/or radiation therapy), with 62.9% of the women undergoing systemic treatment.

#### Individuals after divorce (sample C)

This sample comprised 157 individuals who had gone through a divorce. Almost three out of four participants were women (76.4%), remaining consisted of men (23.6%). The respondents’ average age was 41 years (*M* = 41.29, *SD* = 8.86). More than half of the respondents (54.10%) were residents of large or medium-sized cities. Almost half of the respondents had completed higher education (47.8%), followed by secondary (35.3%), vocational (15.6%), and primary education (1.3%). The vast majority of the respondents (81.1%) were employed. Most respondents reported their current financial status to be average (37.8%) or good (36.5%). Of all the participants, 47.8% had one child, 32.1% had two children, and 7.5% had three or more children, with the remaining 9.4% having no children. The average marriage duration was 11 years (*M* = 10.80, *SD* = 6.80). A total of 47.8% of respondents stated that they had not been involved in any other romantic relationship since their divorce and 32.1% reported that they were in a new romantic relationship, while 18.2% of the individuals had remarried.

### Measures

#### Ego-resiliency

The level of ego-resiliency was measured with the Polish adaptation of the Ego-Resiliency Scale (ER89; Block & Kremen, 1996; adaptation by Kołodziej-Zaleska & Przybyła-Basista, 2018a). The Polish version of the questionnaire (ER89-R12) includes 12 items. Each item has a 4-point response scale (4 = “Applies very strongly”, 1 = “Does not apply at all”). Its psychometric parameters (reliability, stability, validity) are satisfactory (Kołodziej-Zaleska & Przybyła-Basista, 2018a). This revised version (ER89-R12) has a two-factor structure, as does the revised version (ER89-R) proposed by Alessandri et al. (2007; 2012). The Polish version of the scale structure also consists of two factors: Optimal Regulation (OR) (exemplary item: “I quickly get over and recover from being startled”) and Openness to Life Experience (OL) (exemplary item: “I like to take different paths to familiar places”). It is possible to obtain an overall score in the questionnaire, as well as scores for the individual subscales. The Ego-Resiliency Scale was used in all three samples. The internal consistency of this scale was between *α* = 0.802 and *α* = 0.888 (depending on the sample).

#### Psychological well-being (PWB)

The Polish adaptation (Kołodziej-Zaleska & Przybyła-Basista, 2018b) of the Oxford Happiness Questionnaire (OHQ) (Hills & Argyle, 2002) was used to measure the level of psychological well-being defined as life satisfaction, sense of power, and sense of purpose and control in samples A and C. The Polish adaptation (Kossakowska, Kwiatek & Stefaniak, 2013) of the Meaning of Life Questionnaire (Steger et al., 2006) was used to measure search for sense and presence of sense in sample B. The internal consistency was between 0.902 and 0.910. (depending on the sample).

#### Perceived social support (PSS)

The Polish adaptation (Buszman & Przybyła-Basista, 2017) of the Multidimensional Scale of Perceived Social Support (MSPSS) (Zimet et al., 1988) was used in samples A and B. The Polish adaptation (Szlachta, 2009) of the Interpersonal Support Evaluation List (Cohen et al., 1985) was used in sample C. The internal consistency was between 0.893 and 0.946.

#### Perceived stress (PS)

The Polish adaptation (Juczyński & Ogińska-Bulik, 2009) of the Perceived Stress Scale (PSS-10) (Cohen, Kamarck & Mermelstein, 1983) was used to measure of the degree to which situations in one’s life are appraised as stressful in sample A. The Stress Appraisal Questionnaire (SAQ) (Włodarczyk & Wrześniewski, 2010) was used to measure intensity of appraisal as a threat related to a stressful situation in sample C. The internal consistency was between 0.780 and 0.860.

#### Post-traumatic growth (PG)

The Polish adaptation (Ogińska-Bulik & Juczyński, 2010) of the Post-traumatic Growth Inventory (Tedeschi & Calhoun, 1996) was used to measure positive changes that occur as a result of struggling with traumatic events in sample B. The Life Changes Scale (LCS) (Zięba, Wawrzyniak & Świrkula, 2010) was used to measure changes in the functioning of a person suggestive of post-traumatic growth as a result of experiencing a critical life event in sample C. The internal consistency was between 0.800 and 0.913.

#### Self-esteem (SE)

The Polish adaptation (Łaguna, Lachowicz-Tabaczek & Dzwonkowska, 2007) of the Rosenberg Self-Esteem Scale (1965) was used in samples B and C. The internal consistency equaled 0.843.

### Statistical analysis

Statistical analysis of the collected results was carried out using the PS Imago program SPSS Statistic 24 and JASP 0.11.0.1 equipped with the *lavaan* package (Rosseel, 2012). In order to verify the factor structure of the original ER version, a confirmatory factor analysis (CFA) was performed using a DWLS estimator (diagonally weighted least squares, *i.e.*, the estimation procedure for categorical variables with both multivariate normal and non-normal distributions (Mîndrilă, 2010). The DWLS methods have worked well in many conditions, including smaller samples and non-normal data (Rhemtulla, Brosseau-Liard & Savalei, 2012). The CFA was conducted to examine the goodness of fit of the data to the two-factor model proposed by Alessandri, and colleagues (2007). Multigroup confirmatory factor analysis (MGCFA) was carried out in order to demonstrate equivalence of measurement in all samples (Putnick & Bornstein, 2016; Sass, Schmitt & Marsh, 2014). The series of one way ANOVA (ANOVA) were carried out with bootstrapping (*N* = 1, 000), with bias-corrected and accelerated confidence intervals.

In order to determine the level of measurement equivalence in the samples, according to the criteria proposed by Chen (2007), metric invariance be determined if the delta CFI ≤ −0.01, combined with delta RMSEA ≤ 0.015 or with delta SRMR ≤ 0.03. The scalar equivalence occurs when the difference in CFI values for this model compared to the metric equivalence model is not greater than 0.01 combined with the difference in RMSEA values is not greater than 0.015 or delta SRMR is not greater than 0.01.

## Results

**Figure 1:**
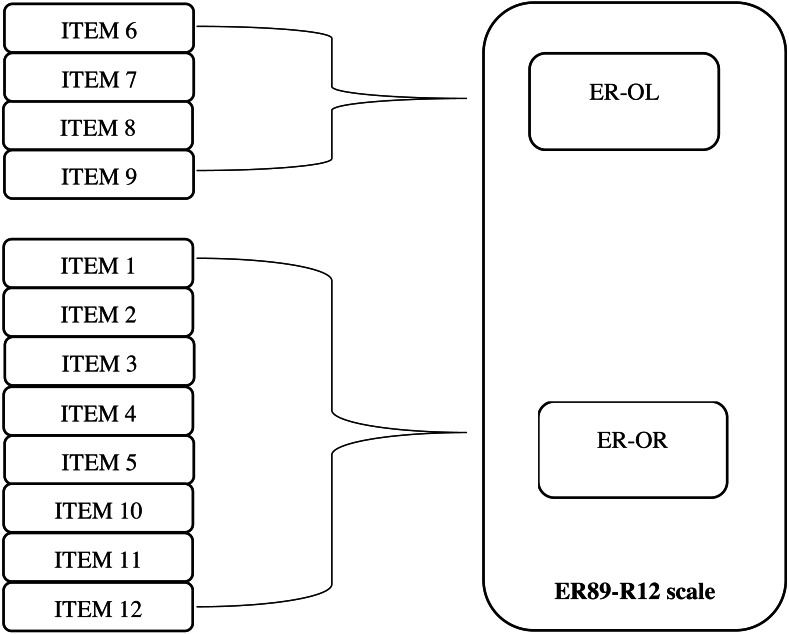
Diagrammatical representation of the structural model of the ER89-R12 Scale. ER, Ego-Resiliency; OR, Optimal Regulation; OL, Openness to Life Experience.

The MGCFA was used to assess the measurement invariance of the ER89-R12 Scale across the three groups. As a first step, we tested the factor structure of the scale within each group separately. This model adopted by Kołodziej-Zaleska & Przybyła-Basista (2018a), shown in Fig. 1, was consistent within two factors: Ego-Resiliency Optimal Regulation (ER-OR) and Ego-Resiliency Openness to Life Experience (ER-OL) proposed Alessandri et al. (2007; 2012).

Before comparing the groups, it is important to make sure that the structure provides good fit for all groups. Thus, the first step is to test whether the proposed two-factor model of ER fits the empirical data from each group. Three CFAs were conducted for sample A, B, and sample C separately. The results show an excellent model fit for each sample, indicating that the two-factor model of ego-resiliency is supported in all samples. The CFA model with the unconstrained factor loadings and intercepts is shown in Table 1.

**Table 1:** Goodness of fit indices for two-factor model among three samples.

Group	*df*	*χ2*	*p*	*RMSEA*	*GFI*	*TLI*	*CFI*
Sample A	53	54.703	0.410	0.016	0.972	0.997	0.997
Sample B	53	43.094	0.832	0.001	0.991	1.000	1.000
Sample C	53	35.036	0.973	0.001	0.987	1.000	1.000

**Notes.**

Sample A: parents of children with Down’s syndrome; sample B: women with breast cancer; sample C: individuals after divorce.

The adequacy of these models can also be determined in relation to the parameter estimates. Standardized factor loadings for the two-factor CFA are included in Table 2.

**Table 2:** Standardized CFA loadings for the two grouping factors of the Ego-Resiliency Scale.

Construct	Item	Sample A *β*	*Sample Bβ*	*Sample Cβ*
OR –Optimal Regulation	ER1	0.351	0.585	0.468
	ER2	0.543	0.682	0.658
	ER3	0.756	0.711	0.748
	ER4	0.546	0.733	0.637
	ER5	0.607	0.707	0.615
	ER10	0.532	0.756	0.759
	ER11	0.710	0.684	0.769
	ER12	0.450	0.583	0.618
OL –Openness to Life Experience	ER6	0.388	0.518	0.555
	ER7	0.745	0.573	0.669
	ER8	0.479	0.677	0.617
	ER9	0.835	0.698	0.828

**Notes.**

All factor loadings are statistically significant (*p* < 0.001); sample A: parents of children with Down’s syndrome; sample B: women with breast cancer; sample C: individuals after divorce.

As the results of the ER Scale are derived from three samples, before they could be compared, an MGCFA was performed to demonstrate the equivalence of the measurement in the three samples (Beaujean, 2014). In the first stage of the study, an analysis of the equivalence of the ER measurements was carried out in the group of parents of children with Down’s syndrome, in the group of women with breast cancer, and in the group of people after divorce. The purpose of these analyses was to show that the ER Scale has comparable psychometric properties, regardless of the type of crisis situation people are facing. We tested for measurement invariance (MGCFA) in order to cross-validate the two-factor model across the three samples; see Table 3 for the fit indices.

**Table 3:** Test of measurement invariance (samples A, B and C).

	*χ* ^2^	df	p	CFI	ΔCFI	SRMR	ΔSRMR	RMSEA	Δ RMSEA
Model 1^*^	133.485	159	0.930	1.000	—	0.060	—	0.000	—
Model 2^**^	200.707	179	0.127	0.995	0.005	0.073	0.013	0.026	0.026
Model 3^***^	218.065	199	0.169	0.995	0.000	0.070	−0.003	0.023	−0.003
Model 4^****^	250.467	223	0.100	0.993	0.002	0.076	0.006	0.026	0.003

**Notes.**

* configural invariance.

** metric invariance

*** scalar invariance

**** full uniqueness MI

Sample A: parents of children with Down’s syndrome; sample B: women with breast cancer; sample C: individuals after divorce.

Four measurement invariance steps were considered: (1) configural, equivalence of model form; (2) metric (weak factorial), equivalence of factor loadings; (3) scalar (strong factorial), equivalence of item intercepts or thresholds; and (4) residual (strict or invariant uniqueness), equivalence of item residuals or unique variances (see: Chen, 2007; Putnick & Bornstein, 2016). As the excellent fit of the two-factor structure had been established independently for each group in the first step, it could be expected that configural invariance would be supported; indeed, this was confirmed by the fit indices, as shown in Table 3. Model 1 provides an excellent fit to the data, indicating that the factorial structure of the construct is equal across groups. As the configural invariance was supported, the factor pattern coefficients were then constrained to be equal to the test for metric invariance. Model 2 had good fit indices (see Table 3). The comparative fit indices (ΔCFI = 0.005) indicate that the viability of constraining the factor loading was the same across samples (ΔRMSEA is 0.026, but ΔSRMR is good, it equals 0.013). The scalar invariance model (Model 3) and the residual invariance model (Model 4) provide an excellent fit to the data. Support for scalar invariance indicates that the latent means can be meaningfully compared across samples. Support for residual invariance indicates that the sum of specific variance and error variance is similar across groups. The analysis above supports the measurement invariance of the two-factor model of ego-resiliency across the three groups.

The obtained results indicate the scalar equivalence of the measurements in both samples (the CFI value for the scalar equivalence model was 0.995 compared to the CFI value for the metric equivalence model, which was also 0.995; ΔSRMR was not greater than 0.01, the RMSEA difference for the compared models was 0.003). This result enables the conclusion that the ER Scale maintains comparable psychometric properties in studies of parents of children with Down’s syndrome, women with breast cancer and people after divorce. It is therefore possible to compare the mean results for all three groups.

The one-way ANOVA carried out at a later stage of the study for comparative analysis of the three groups shows statistically significant differences for the main ER score (F(3, 546) = 3.02; *p* < 0.05) and for Optimal Regulation (F(3, 546) = 3.29; *p* < 0.05). With an in-depth analysis including assessment between the three groups, the differences remained significant between the following groups: ER: sample A and sample B (95% CI [3.38; −0.008]); OR: sample A and sample B (95% CI <−2.41; −0.03>). The highest ER was found in the women with breast cancer (*M* = 35.26; *SD* = 6.78), followed by the divorced individuals (*M* = 35.09; *SD* = 6.71), and the lowest ER was found in parents of children with Down’s syndrome (*M* = 33.53; *SD* = 6.25). See Table 4 for details.

In the next stage of the analysis, the correlation coefficients between ER and the other tested variables were calculated in order to assess the construct validation. The Pearson correlation was calculated between the individual mean scores on the ER89-R12 and the some measures of mental health: psychological well-being, perceived social support, post-traumatic growth, personality traits like self-esteem, and perceived stress. As expected, higher levels of ego-resiliency were strongly associated with psychological well-being. The size of the coefficient was fairly high in each sample. Ego-resiliency showed a positive correlation with perceived social support, self-esteem, and post-traumatic growth. Furthermore, ego-resiliency was negatively correlated with perceived stress (Table 5).

**Table 4:** Descriptive statistics for the three samples.

	Group	*N*	*Cronbach’salpha*	*M*	*SD*	*MIN*	*MAX*	*F(df)*	*p*	*η* ^2^
ER	Sample A	126	0.819	33.53	6.25	17.00	48.00	3.029 (546)	0.049	0.01
	Sample B	269	0.887	35.26	6.78	13.00	48.00			
	Sample C	154	0.879	35.09	6.71	13.00	48.00			
OR	Sample A	126	0.790	22.34	4.39	13.00	32.00	3.298 (546)	0.038	0.01
	Sample B	269	0.873	23.60	4.80	8.00	32.00			
	Sample C	154	0.861	23.55	4.73	9.00	32.00			
OL	Sample A	126	0.692	11.18	2.80	4.00	16.00	1.333 (546)	0.268	–
	Sample B	269	0.708	11.65	2.60	4.00	16.00			
	Sample C	154	0.758	11.54	2.72	4.00	16.00			

**Notes.**

ER general score OR Optimal Regulation OL Openness to Life Experience

sample A: parents of children with Down’s syndrome; sample B: women with breast cancer; sample C: individuals after divorce.

**Table 5:** Construct validity of the ER89-12R individual total score in Samples A, B and C.

Sample A	PWB	PSS	PS	—	—
ER	0.483^**^	0.245^**^	−0.455^**^	—	—
Sample B	PWB	PSS	—	PG	SE
ER	0.344^*^	0.250^**^	—	0.273^**^	0.280^**^
Sample C	PWB	PSS	PS	PG	SE
ER	0.442^**^	0.436^**^	−0.378^**^	0.404^**^	0.674^**^

**Notes.**

PWB Psychological well-being PSS Perceived social support PS Perceived stress PG Post-traumatic growth SE Self-esteem

* *p* < 0.05

** *p* < 0.01

sample A: parents of children with Down’s syndrome; sample B: women with breast cancer; sample C: individuals after divorce.

## Discussion

The aim of our work was to confirm the structure of the tool in groups of people facing various psychological crises. For this purpose, a measurement invariance analysis was used. Measurement invariance assesses the psychometric equivalence of a construct across groups or measures and shows whether the construct has the same meaning in these groups or repeated measures. The conducted measurement equivalence analysis, composed of several levels, showed that the representatives of the various groups we studied have the same understanding of the ego-resiliency construct. They assign the same weighting to the test items of the ER89-R12 Scale in their responses, meaning that the answers obtained in all three groups are comparable with one another. In our research four levels of equivalence were tested: configural, metric, scalar and residual invariance. Therefore, as the presented results of the analysis show, the ER89-R12 tool is an accurate measure of ego-resiliency in groups of people facing various crises. In other words, the latent construct is measured identically across groups and, as in the Italian version of the tool (Alessandri et al., 2007; Alessandri et al., 2012), it includes two subscales: optimal regulation and openness to life experience. The presented results of the analysis make it possible to compare the mean results on the ER89-R12 Scale and its subscales between the studied smples. The conducted comparative analysis showed that the level of ego-resiliency differed depending on the studied group. However, differences between groups were small referring to effect size. Despite the differences in the ego-resiliency levels between the groups, the analysis of the construct validity of the ER89-R12 in the three groups indicated the same patterns of dependencies between variables. The results show that ego-resiliency was positively correlated with measures of mental health: psychological well-being, post-traumatic growth, self-esteem and perceived social support, and negatively correlated with perceived stress in each of the analysed groups. The correlations are consistent with the predictions, theoretical assumptions and results of other similar analyses (Lepore & Revenson, 2006). The results confirm the conclusion that ego-resiliency is a construct related to the notions of adjustment, maladjustment and psychological health (Alessandri et al., 2012; Block & Block, 1980; Letzring, Block & Funder, 2005; Vecchione et al., 2010). As Block & Kremen (1996) noted, individuals with high ego-resiliency are better adapted and have the ability to deal more effectively with both changing everyday events and difficult situations than those with low ego-resiliency. Research shows that ego-resiliency is a key personal resource that might be able to buffer the detrimental effects of daily stressors on individuals’ negative emotional inertia (Alessnadri et al., 2020). Ego-resiliency explains the functioning of people who cope well with stress (Kaczmarek, 2011). Therefore, it can also be expected that ego-resiliency is related to those aspects of functioning that enable effective adaptation, coping, flexibility, and a committed and active approach to the world (Alessandri et al., 2007).

Ego-resiliency is a trait that plays a protective role in the adaptation process (Ilska, Brandt-Salmeri & Kołodziej-Zaleska, 2020; Kołodziej-Zaleska & Przybyła-Basista, 2020; Izydorczyk et al., 2018) Used in conjunction with the variables determining psychological well-being, support, coping with stress and trauma, and other personality traits which are protective factors in the process of adaptation to crises, the Ego-Resiliency Scale allows for a deeper understanding of how ego-resiliency works. Ego-resiliency can be considered a potential factor facilitating the process of adaptation (Shin et al., 2019). According to current knowledge, individuals with a high level of ego-resiliency find it easier to adapt to changing circumstances (Block & Kremen, 1996; Vecchione et al., 2010), which is important for people facing crises. Ego-resiliency can help people deal more quickly with loss, grief, guilt, sadness and other negative feelings, and focus on adaptive flexibility and coping effectively in life.

Research studies on resilience, understood as a personality trait, in life crisis situations such as post-divorce, illness or a child’s disability are not carried out frequently (Caples et al., 2018; Frisby et al., 2012; Izydorczyk et al., 2018; Perrig-Chiello, Hutchison & Morselli, 2015; Seiler & Jenewein, 2019; Van Riper, 2007). Bonanno, Westphal & Mancini (2011, 2012) discuss whether resiliency (understood as an individual’s disposition) plays a very important role in the process of adapting to various crisis events but rarely appears in research in this context. Klohnen (1996), meanwhile, notes that too little attention is paid to resilience, understood as a variable, in the field of individual differences (see also Ilska, Brandt-Salmeri & Kołodziej-Zaleska, 2020).

Most of the large-scale studies using the Ego-Resiliency Scale have been conducted with adolescents and young adults in the general population (Alessandri et al., 2014; Caprara et al., 2014; Block & Kremen, 1996; Letzring, Block & Funder, 2005; Vecchione et al., 2010). The analysis conducted here confirms that the tool can be used unreservedly in research with older or middle-aged people and, importantly, people who are struggling with various adversities. Due to the variety of crises experienced by the respondents (crisis related to a child’s illness, crisis related to losing one’s own health and struggling with a terminal illness, and family crisis related to the experience of divorce), it can also be assumed that the ER89-R12 will be an adequate measurement tool in the case of other life crises, allowing the results of people with various difficult life experiences to be compared.

An additional and interenting result of the analysis carried out is the difference in the level of ego-resiliency in the group of parents of children with Down’s syndrome. Compared to other groups the level of ego-resiliency was lower. Looking at the distinct components, the differences relate to optimal regulation, but they do not occur in the area of openness to life experience, which supports the process of accepting the state of affairs, taking action, and flexible focus on goals. Perhaps parents of children with Down’s syndrome have difficulty in adapting to the role of parents of a child with a mental disability. They experience a higher level of stress than parents of healthy children (Baker et al., 2003). In the light of the presented results, in the case of parents of children with Down’s syndrome, ego-resiliency is weaker than in the group of people facing a crisis related to their own terminal illness. Adequate modulation of emotion, flexible adaptation of responses, and readiness to face new and unexpected situations are of key importance in enduring cancer. Ego-resiliency promotes the mobilization of the patient to take various remedial actions; it may act as a resource that increases tolerance to the ambivalence and ambiguity that are part of the patient’s reality. For breast cancer patients, ego-resiliency has a regulatory function and may also facilitate the adoption of an open attitude towards the challenges accompanying the disease. This trait is a kind of meta-resource that optimizes the relationship between personality and environmental variables and remains superior to other resources. It provides flexibility as well as a greater ability to search for meaning in what is happening (Farkas & Orosz, 2015). It aids optimal adaptation, especially in the experience of somatic disease (Park, 2010).

Coping proactively with the effects of a crisis by mobilizing resources appears to be more likely, but also more effective, when the disease affects oneself rather than one’s child. It seems that a child’s disability could be a factor that disturbs the world of values, emotional balance and sense of meaning to a greater extent than a cancer diagnosis. Being the parent of a child with Down’s syndrome is undoubtedly a crisis of a permanent nature. In the case of women with breast cancer or divorced people, the crisis may be characterized by a narrow period of time, often limited to coping with the acute effects of the stressors experienced. Having a disabled child, on the other hand, is a crisis with an unlimited time spectrum. Moreover, it is a crisis the impact of which extends not only to the adult, but above all to the child. It can be assumed that this disturbs the coherent image of a valuable ‘I’, and the hope for favourable circumstances in the future, to a greater extent. The birth of a child with a disability usually induces in parents a sense of being trapped with no possibility of escape (Będkowska-Heine, 2007). According to researchers (Kaczmarek, Sęk & Ziarko, 2011), people with lower levels of ego-resiliency tend to focus on failures, have little emotional diversity and often suffer from a lack of meaning in life. Furthermore, the lower level of ego-resiliency of parents of children with Down’s syndrome may be related to higher levels of perceived stress and lower levels of psychological well-being (Kózka & Przybyła-Basista, 2017).

On the other hand, some research has provided support for the belief that many families of children with Down’s syndrome respond to challenges with resilience (Van Riper, 2007). According to certain studies, families of children with Down’s syndrome can adapt and become resilient; factors found to positively influence this process include family hardiness and affirming family communication (Caples et al., 2018). These studies, however, focus on family resilience, indicating the aspects of family life that enable adaptation to the circumstances of the child’s illness. They are much more about resilience as a process than a personality trait. They provide no information about the parents’ individual resources.

This study is an empirical attempt to examine the measurement invariance of the ego-resiliency measure across diverse groups of individuals experiencing life crises. Assessing the questionnaire’s capacity to measure the same construct in different contexts is important from theoretical and practical perspectives. Our findings suggest that the interpretation of items on the scale did not differ among individuals facing life crises. It means that individuals from the three groups interpret the items in an equivalent manner (Hussey & Hughes, 2020). The scores on the scale are likely to measure the same latent variable in a comparable way, regardless of the groups to which the individuals belong. Moreover, our findings show that ER89-R12 has essential properties: good test-retest reliability and clear factor structure in line with the previous validation studies on the ER89-R (Alessandri et al., 2007; Alessandri et al., 2012; Vecchione et al., 2010). Our results can be helpful for researchers investigating the predictors of resilience during the adaptation process after the crisis. Confidence in the comparability and replicability of research findings is a fundamental pillar for progress in research (Hussey & Hughes, 2020). Our analyzes also showed that further studies on the invariant and replicability of the scale structure in various populations, both general and clinical populations, are necessary. Our findings support the validity of the two-factor model of ego-resiliency construct among individuals facing a crisis. We recommend using the ER89-R-12 in research projects because of its good psychometric properties, easy administration, and applicability in the life-crises context.

## Limitations

There are several limitations of the presented study. First, women predominated in all studied samples. It would be helpful in future research to include more men to evaluate the validity of the ER89-R12 Scale and measure invariance across gender. Second, due to the convenient samples used in the study, the generalization of these findings should be conducted with caution. Third, in our research, we did not always use the same instrument to measure psychological well-being and related to its constructs. So the Oxford Happiness Questionnaire (OHQ) was administered in two samples (among divorced people within sample A and parents of children with Down syndrome in sample C). Still, the Meaning in Life Questionnaire was distributed only in the study of women with breast cancer, sample B. We are aware that the hedonistic view of happiness or subjective view of well-being (*e.g.*, Diener, 2000) differs from the eudaimonic view of well-being, emphasizing personal potential, virtue, and meaningful life (Ryff & Singer, 1998). Thus, the measurement tools of the OHQ (Hills & Argyle, 2002) and MLQ (Steger, Oishi & Kashdan, 2009) are not the same. However, in the broadest sense, the term “well-being” refers to people’s optimal functioning and positive experiences (Steger & Samman, 2012) and includes both a sense of subjective happiness and life meaning (Lopez, Pedrotti & Snyder, 2015). Therefore, we use the term well-being when describing the results obtained in these questionnaires.

Our study was part of a larger project on the determinants of adaptation effectiveness in the face of critical life events. One of the research tasks was to test the role of ego-resiliency. In this paper, we present only the part of our study that deals with validating the ER89-R12 measurement tool and its invariance. Ego-resiliency was measured with the ER89-R12 questionnaire in all samples. But the instruments measuring stress, perceived social support or growth after trauma/ crisis were already somewhat more differentiated and tailored to the needs of the study in the specific groups. It seems important that the correlations between ego-resiliency and the analysed constructs indicate the similarity of the patterns of dependencies between the distinct groups. Therefore, future studies need to be repeated using the same measurement tools. There are also limitations on the intergroup comparisons due to the group sizes being large enough that detected differences may be treated rather little in size.

##  Supplemental Information

10.7717/peerj.14499/supp-1 Supplemental Information 1 Ego-resiliency (data, sample A, B, C) The 12 variables are the next statements in the ego-resiliency questionnaire. Group means the first, second, or third group of respondents described in the article. The next column is the overall result, and the next are the results for each subscale of the questionnaire. They are described in the variables tab.

## Additional Information and Declarations

### Data Availability

The following information was supplied regarding data availability: The raw data is available in the Supplementary File.

## References

[ref-1] Alessandri G, Luengo Kanacri BP, Eisenberg N, Zuffianò A, Milioni M, Vecchione M, Caprara GV (2014). Prosociality during the transition from late adolescence to young adulthood: the role of effortful control and ego-resiliency. Personality and Social Psychology Bulletin.

[ref-2] Alessandri G, Vecchione GM, Steca P, Caprara MG, Capara GV (2007). A revised version of Kremen and Block’s Ego Resiliency Scale in an Italian sample. TPM.

[ref-3] Alessandri G, Vecchione M, Capara G, Letzring TD (2012). The Ego resiliency scale revised: a crosscultural study in Italy, Spain, and the United States. European Journal of Psychological Assessment.

[ref-4] Alessnadri G, De Longis E, Eisenberg N, Hobfoll SE (2020). A multilevel moderated mediational model of the daily relationships between hassles, exhaustion, ego-resiliency and resulting emotional inertia. Journal of Research in Personality.

[ref-5] Arat G (2014). A critical systematic review of studies regarding resilience in Turkey: a call for the socio-ecology of resilience perspective. International Journal of Social Work and Human Services Practice.

[ref-6] Baker BL, McIntyre LL, Blacher J, Crnic K, Edelbrock C, Low C (2003). Pre-school children with and without developmental delay: behavior problems and parenting stress over time. Journal of Intellectual Disability Research.

[ref-7] Beaujean AA (2014). Latent variable modeling using R: a step-by-step guide.

[ref-8] Będkowska-Heine V, Cytowska B, Winczura B (2007). Wpływ przewlekłej choroby dziecka na funkcjonowanie w roli ojca [The impact of the child’s chronic condition on functioning in the role of father]. Dziecko chore. Zagadnienia biopsychiczne i pedagogiczne [The ill child. Biopsychical and pedagogical questions.

[ref-9] Block JH, Block J, Collins WWA (1980). The role of ego-control and ego-resiliency in the origination of behavior.

[ref-10] Block JL, Kremen AM (1996). IQ and ego-resiliency: conceptual and empirical connections and separateness. Journal of Personality and Social Psychology.

[ref-11] Bonanno GA (2004). Loss, trauma, and human resilience: have we underestimated the human capacity to thrive after extremely aversive events?. American Psychologist.

[ref-12] Bonanno GA, Mancini AD (2012). Beyond resilience and PTSD: mapping the heterogeneity of responses to potential trauma. Psychological Trauma: Theory, Research, Practice, and Policy.

[ref-13] Bonanno GA, Westphal M, Mancini AD (2011). Resilience to loss and potential trauma. Annual Review of Clinical Psychology.

[ref-14] Brandt-Salmeri A (2020). Rak piersi –wyznaczniki pozytywnych zmian [Breast cancer - determinants of positive changes].

[ref-15] Buszman K, Przybyła-Basista H (2017). Polska adaptacja Wielowymiarowej Skali Spostrzeganego Wsparcia Społecznego [The Polish adaptation of Multidimensional Scale of Perceived Social Support]. Polskie Forum Psychologiczne.

[ref-16] Caples M, Martin A-M, Dalton C, Marsh L. Savage, E, Knafl G, Van Riper M (2018). Adaptation and resilience in families of individuals with down syndrome living in Ireland. British Journal of Learning Disabilities.

[ref-17] Caprara GV, Kanacri BPL, Gerbino M, Zuffianò A, Alessandri G, Vecchio G, Caprara E, Pastorelli C, Bridglall B (2014). Positive effects of promoting prosocial behavior in early adolescence: evidence from a school-based intervention. International Journal of Behavioral Development.

[ref-18] Chen FF (2007). Sensitivity of goodness of fit indexes to lack of measurement invariance. Structural Equation Modeling.

[ref-19] Cohen S, Kamarck T, Mermelstein R (1983). A global measure of psychological stress. Journal of Health and Social Behavior.

[ref-20] Cohen S, Memelstein R, Kamarck T, Hoberman H, Sarason IG, Sarason B (1985). Measuring the functional components of social support. Social support: theory, research and application.

[ref-21] Diener E (2000). Subjective well-being: the science of happiness and a proposal for a national index. American Psychologist.

[ref-22] Farkas D, Orosz G (2015). Ego-resiliency reloaded: a three-component model of general resiliency. PLoS ONE.

[ref-23] Fletcher D, Sarkar M (2013). Psychological resilience a review and critique of definitions, concepts, and theory. European Psychologist.

[ref-24] Frisby B, Booth-Butterfield M, Dillow MR, Martin MM, Weber D (2012). Face and resilience in divorce: the impact on emotions, stress, and post-divorce relationships. Journal of Social and Personal Relationships.

[ref-25] Hills P, Argyle M (2002). The oxford happiness questionnaire: a compact scale for measurement of psychological well-being. Personality and Individual Differences.

[ref-26] Hussey I, Hughes S (2020). Hidden invalidity among 15 commonly used measures in social and personality psychology. Advances in Methods and Practices in Psychological Science.

[ref-27] Ilska M (2020). Ciaża prawidłowa i wysokiego ryzyka. Dobrostan psychiczny i jego wyznaczniki [Normal and high-risk pregnancy. Mental well-being and its determinants].

[ref-28] Ilska M, Brandt-Salmeri A, Kołodziej-Zaleska A (2020). Effect of prenatal distress on subjective happiness in pregnant women: the role of prenatal attitudes towards maternity and ego-resiliency. Personality and Individual Differences.

[ref-29] Ilska M, Przybyła-Basista H (2020). The role of partner support, ego-resiliency, prenatal attitudes towards maternity and pregnancy in psychological well-being of women in high-risk and low-risk pregnancy. Psychology, Health & Medicine.

[ref-30] Izydorczyk B, Kwapniewska A, Lizinczyk S, Sitnik-Warchulska K (2018). Psychological resilience as a protective factor for the body image in post-mastectomy women with breast cancer. International Journal of Environmental Research and Public Health.

[ref-31] Juczyński Z, Ogińska-Bulik N (2009). Narzędzia pomiaru stresu i radzenia sobie ze stresem [Tools for measuring stress and coping with stress].

[ref-32] Kaczmarek Ł (2011). Skala Sprężystości Psychicznej –polska adaptacja Ego Resiliency Scale [Skala Sprężystości Psychicznej –the Polish adaptation of Ego Resiliency Scale]. Czasopismo Psychologiczne.

[ref-33] Kaczmarek Ł, Sęk H, Ziarko M (2011). Sprężystość psychiczna i zmienne pośredniczące w jej wpływie na zdrowie [Resilience and variables mediating its impact on health]. Przegląd Psychologiczny.

[ref-34] Klohnen EC (1996). Conceptual analysis and measurement of the construct of Ego-Resiliecy. Journal of Personality and Social Psychology.

[ref-35] Kołodziej-Zaleska A (2019). Życie po rozwodzie. Zróżnicowany process adaptacji. [Life After Divorce: Variation in the Post-Divorce Adjustment].

[ref-36] Kołodziej-Zaleska A, Przybyła-Basista H (2018a). Ego-resiliency jako zasób osobisty –narzędzie pomiaru i jego wykorzystanie w badaniach interdyscyplinarnych, [Ego-resiliency as a personal resource –an assessment instrument and its use in interdisciplinary research]. Psychological Journal.

[ref-37] Kołodziej-Zaleska A, Przybyła-Basista H (2018b). Dobrostan psychiczny i jego pomiar za pomocą polskiej wersji, Oksfordzkiego Kwestionariusza Szczęścia. [Psychological happiness and its measurement with Polish version of Oxford Happiness Questionnaire]. Psychological Journal.

[ref-38] Kołodziej-Zaleska A, Przybyła-Basista H (2020). The role of ego-resiliency in maintaining post-divorce well-being in initiators and non-initiators of divorce. Journal of Divorce & Remarriage.

[ref-39] Kossakowska M, Kwiatek P, Stefaniak T (2013). Sens w życiu. Polska wersja kwestionariusza MLQ (Meaning in life questionnaire (meaning in life. The Polish version of meaning in life questionnaire MLQ). Psychologia Jakości Życia.

[ref-40] Kowalczyk M, Orzechowska A, Talarowska M, Zboralski K, Macander M, Truszczyński O, Gałecki P (2015). Resilience in the process of coping with traumatic situations among pilots in the foreign missions. The Polish Journal of Aviation Medicine, ioengineering and Psychology.

[ref-41] Kózka A, Przybyła-Basista H (2017). Ego-resiliency and parental satisfaction among parents of children with down syndrome. The New Educational Review.

[ref-42] Kózka A, Przybyła-Basista H (2018). Perceived stress, ego-resiliency, and relational resources as predictors of psychological well-being in parents of children with Down syndrome. Health Psychology Report.

[ref-43] Łaguna M, Lachowicz-Tabaczek K, Dzwonkowska I (2007). Skala Samooceny SES Morrisa Rosenberga –polska adaptacja metody [The Self-esteem Scale SES Morris Rosenberg –Polish adaptation of the method]. Psychologia Społeczna.

[ref-44] Lepore SJ, Revenson TA, Calhoun LG, Tedeschi RG (2006). Resilience and posttraumatic growth: recovery, resistance, and reconfiguration. Handbook of posttraumatic growth: research & practice.

[ref-45] Letzring TD, Block J, Funder DC (2005). Ego-control and ego-resiliency: generalization of self-report scales based on personality descriptions from acquaintances, clinicians, and the self. Journal of Research in Personality.

[ref-46] Lopez SJ, Pedrotti JT, Snyder CR (2015). Positive psychology.

[ref-47] Luthar SS, Cicchetti D, Cohen DJ (2015). Resilience in development: a synthesis of research across five decades. Developmental psychopathology: risk, disorder, and adaptation, Volume Three, Second Edition.

[ref-48] Luthar SS, Cicchetti D, Becker B (2000). The construct of resilience: a critical evaluation and guidelines for future work. Child Development.

[ref-49] Menesini E, Fonzi A (2005). Strategie di coping e caratteristiche di resilienza in adolescenza. Psicologia Clinica Dello Sviluppo.

[ref-50] Mîndrilă D (2010). Maximum Likelihood (ML) and diagonally weighted least squares (DWLS) estimation procedures: a comparison of estimation bias with ordinal and multivariate non-normal data. International Journal of Digital Society.

[ref-51] Ogińska-Bulik N (2013). Pozytywne skutki doswiadczeń traumatycznych czyli kiedy łzy zamieniają się w perły’ [Positive effects of traumatic experiences when tears turn into pearls].

[ref-52] Ogińska-Bulik N, Juczyński Z (2010). Rozwój potraumatyczny - charakterystyka i pomiar. [Posttraumatic growth –characteristics and measurement]. Psychiatria.

[ref-53] Ong AD, Bergeman CS, Bisconti TL, Wallace KA (2006). Psychological resilience, positive emotions, and successful adaptation to stress in later life. Journal of Personality and Social Psychology.

[ref-54] Park CL (2010). Making sense of the meaning literature: an integrative review of meaning making and its effects on adjustment to stressful life events. Psychological Bulletin.

[ref-55] Perrig-Chiello P, Hutchison S, Morselli D (2015). Patterns of psychological adaptation to divorce after a longterm marriage. Journal of Social and Personal Relationships.

[ref-56] Putnick DL, Bornstein MH (2016). Measurement invariance conventions and reporting: the state of the art and future directions for psychological research. Developmental Review.

[ref-57] Pyszkowska A (2020). Personality predictors of self-compassion, ego-resiliency and psychological flexibility in the context of quality of life. Personality and Individual Differences.

[ref-58] Quinney DM, Fouts GT (2004). Resilience and divorce adjustment in adults participating in divorce recovery workshops. Journal of Divorce & Remarriage.

[ref-59] Rhemtulla M, Brosseau-Liard PÉ, Savalei V (2012). When can categorical variables be treated as continuous? A comparison of robust continuous and categorical SEM estimation methods under suboptimal conditions. Psychological Methods.

[ref-60] Rosenberg M (1965). Society and the adolescent self-image.

[ref-61] Rosseel Y (2012). https://users.ugent.be/yrosseel/lavaan/lavaanIntroduction.pdf.

[ref-62] Ryff CD, Singer BH (1998). The contours of positive human health. Psychological Inquiry.

[ref-63] Sass DA, Schmitt TA, Marsh HW (2014). Evaluating model fit with ordered categorical data within a measurement invariance framework: a comparison of estimators. Structural Equation Modeling.

[ref-64] Seiler A, Jenewein J (2019). Resilience in cancer patients. Frontiers in Psychiatry.

[ref-65] Shin YC, Kim SM, Kim H, Min KJ, Yoo SK, Kim EJ, Jeon SW (2019). Resilience as a protective factor for depressive mood and anxiety among Korean employees. Journal of Korean Medical Science.

[ref-66] Steger MF, Frazier P, Oishi S, Kaler M (2006). The meaning in life questionnaire: assessing the presence of and search for meaning in life. Journal of Counseling Psychology.

[ref-67] Steger MF, Oishi S, Kashdan TB (2009). Meaning in life across the life span: levels and correlates of meaning in life from emerging adulthood to older adulthood. Journal of Positive Psychology.

[ref-68] Steger MF, Samman E (2012). Assessing meaning in life on an international scale: psychometric evidence for the meaning in life questionnaire-short form among Chilean households. International Journal of Wellbeing.

[ref-69] Szlachta E (2009). Próba adaptacji i walidacji polskiej wersji The Interpersonal Support Evaluation List (ISEL) –Kwestionariusz Spostrzeganego Wsparcia Społecznego. [The adaptation and preliminary validation of the Polish version of The Interpersonal Support Evaluation List]. Przegląd Psychologiczny.

[ref-70] Tedeschi RG, Calhoun LG (1996). The post-traumatic growth inventory: measuring the positive legacy of trauma. Journal of Trauma and Stress.

[ref-71] Tugade MM, Fredrickson BL (2004). Resilient individuals use positive emotions to bounce back from negative emotional experiences. Journal of Personality and Social Psychology.

[ref-72] Van Riper M (2007). Families of children with down syndrome: responding to a change in plans with resilience. Journal of Pediatric Nursing.

[ref-73] Vanderbleek E, Gilbet K (2018). Too much versus too little control: the etiology, conceptualization, and treatment implications of overcontrol and undercontrol. Radically Open.

[ref-74] Vecchione M, Alessandri G, Barbaranelli C, Gerbino M (2010). Stability and change of ego resiliency from late adolescence to young adulthood: a multiperspective study using the ER89-R Scale. Journal of Personality Assessment.

[ref-75] Windle J, Bennett KM, Noyes J (2011). A methodological review of resilience measurement scales. Health and Quality of Life Outcomes.

[ref-76] Włodarczyk D, Wrześniewski K (2010). Kwestionariusz Oceny Stresu (KOS), [The Stress Appraisal Questionnaire]. Przegląd Psychologiczny.

[ref-77] Zięba M, Wawrzyniak M, Świrkula M (2010). Skala Zmian Życiowych - narzędzie do pomiaru skutków krytycznych zdarzeń [Life-changes scale - measure of consequences of critical life events]. Psychologia Jakości Życia.

[ref-78] Zimet GD, Dahlem NW, Zimet SG, Farley GK (1988). The multidimensional scale of perceived social support. Journal of Personality Assessment.

